# Non-Surgical Endodontic Management of Type II Dens Invaginatus with Closed and Open Apex 

**DOI:** 10.22037/iej.v12i4.18078

**Published:** 2017

**Authors:** Hugo Plascencia, Mariana Díaz, Bertram Ivan Moldauer, Mario Uribe, Eddy Skidmore

**Affiliations:** a *Endodontic Postgraduate Program, CUCS-CUAltos, University of Guadalajara, México*;; b *Adjunct Assistant Professor in Endodontics and Surgical Course Co-Director, Nova Southeastern University, College of Dental Medicine, Fort Lauderdale, Florida, USA;*; c *Endodontic Postgraduate Program, CUCS, University of Guadalajara, México;*; d *Department of Endodontics, Nova Southeastern Universitiy College of Dental Medicine, Fort Lauderdale, Florida, USA*

**Keywords:** Calcium Hydroxide, Close Apex, Dens Invaginatus, Open Apex, Revascularization

## Abstract

Dens invaginatus (DI) is a developmental anomaly that poses a significant challenge to the clinician if endodontic treatment is required. The type II (as per Oehlers) form exhibits complex internal anatomy and is frequently associated with incomplete root and apex formation. The purpose of this study is to present two cases of type II DI in the maxillary lateral incisors. In the first case, non-surgical endodontic therapy was performed utilizing calcium hydroxide as an intracanal dressing, showing significant periapical healing of the apical radiolucent area at the six month follow-up. In the second case, the development of the root and apex were affected by pulp necrosis, and the revascularization procedure was performed. Complete resolution of the pre-existing apical radiolucency, apical closure, thickening of the root canal walls, and increase in root length, after 32 months was observed. Early detection of teeth with DI type II and proper exploration of their internal anatomy are key factors for their successful management. As demonstrated in this report, conservative non-surgical endodontic treatment should be the first line of treatment for these cases. The use of revascularization protocols in teeth that develop pulp necrosis and exhibit early stage of root development could be a better alternative than traditional apexification techniques.

## Introduction

Dens invaginatus (DI), also known as dens in dente or invaginated odontoma, is a developmental anomaly resulting from the invagination of the enamel organ into the dental papilla during the soft tissue stage of development [1, 2]. As the hard tissues are formed, the invaginated enamel organ leads to the formation of small tooth within the future pulp chamber [3, 4]. Although the etiology of DI is unclear, this anomaly can affect any tooth, with significant predilection for the maxillary lateral incisors [5, 6].

Three forms of DI have been described by Oehlers (1957) [7] according to the radiographic extent of the invagination. Type I represents an enamel-lined minor form occurring within the confines of the crown, but not extending beyond the cemento-enamel junction. In type II, the invagination invades the root and remains confined as a blind sac. Type III is the form of DI in which the invagination penetrates through the root, perforating at the apical area. Type III DIs have a second foramen in the periodontal (type III a) or apical (type III b) areas. 

In the Oehlers type II DI, the invagination is enamel-lined and extends into the pulp chamber (with variation in shape and depth), but remains within the root canal with no communication with the periodontal ligament. However, the invagination allows entry of irritants into the pulp space through a thin hypo-mineralized enamel and dentin layer, thus causing early necrosis of pulp tissue before the completion of root development. Treatment of DI is dependent upon the specific time of detection as well as the severity and extension of the malformation. Although the management of certain cases of DI type III could be limited to treatment of only one of the root canals [8], type II forms require the attention of both the main canal and the invagination. In many cases, the anatomical location of the invagination may lead to a corono-radicular obstruction, which complicates proper chemo-mechanical disinfection and filling of the main canal [9]. Given the morphological complexities of these cases, many teeth had to be extracted [10]. However, recent advances in technology such as microscopy, ultrasonics, and revascularization techniques have made possible the successful management of these types of malformations in a more predictable manner [11, 12]. The objective of this report is to describe the clinical management of two distinct maxillary lateral incisors with type II DI, one with a closed and one with an open apex.

## Case Report

**Figure 1:**
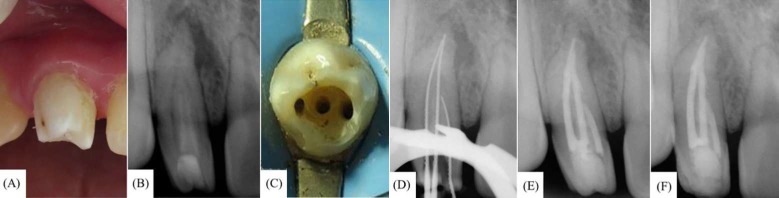
*A)* Crown of maxillary right lateral incisor exhibits a cone-shaped morphology; *B)* Pre-operative radiograph showing Oehlers type II dens invaginatus (DI); *C)* Access cavity depicting three canal orifice entrances; *D)* Working length determination; *E)* Post-operative radiograph; *F)* Follow-up at six months

***Case 1***: A 15-year-old female presented to the Department of Endodontics at University of Guadalajara in México with a chief complaint of upper right lateral anterior tooth having a weird shape. The patient was in good general health. Extra-oral examination revealed no significant findings. Intra-oral examination revealed a “cone”-shaped maxillary right lateral incisor with a deep anatomic pit on the palatal surface (Figure 1A). The tooth was asymptomatic to percussion and did not respond to thermal and electric sensitivity tests. The periapical radiograph revealed a type II DI and periapical pathology (Figure 1B). An endodontic diagnosis of pulp necrosis with asymptomatic apical periodontitis was made. Endodontic treatment was started immediately. The area was anesthetized with 1.8 mL 2% lidocaine with 1:100000 epinephrine (Alphacaine 100; DFL, Rio de Janeiro, RJ, Brazil). After dental dam isolation and gaining access into the pulp chamber, with the aid of magnification throughout the treatment, three distinctly separate areas of pulp tissue were found (Figure 1C). Once determination of working lengths (Figure 1D), biomechanical shaping was performed with rotary and hand instrumentation up to a size #60 K-file. Then, the tooth was irrigated with 3% NaOCl and ultrasonic activation for one min, followed by a final rinse of 10 mL of 17% ethylenediaminetetraacetic acid (EDTA). Calcium hydroxide paste was applied as an intracanal dressing, and the access cavity was temporarily sealed. One week later, the patient returned without any symptoms. At this appointment, the tooth was asymptomatic to percussion and the soft tissues were within normal limits. After dental dam isolation, access was obtained, and the canals were irrigated and re-instrumented using the same protocol applied during the first visit. The canals were dried with paper points and filled by warm vertical condensation of gutta-percha and AH-Plus sealer (Dentsply Tulsa Dental, Tulsa, OK, USA). The canals were then backfilled with an injection-molded thermo plasticized gutta-percha delivery system (Obtura System II, SybronEndo, Coppell, TX, USA) (Figure 1E). At the six month follow-up, the patient reported no symptoms, the tooth was asymptomatic to percussion, and the labial mucosa was not tender to palpation. The radiograph showed significant periapical healing with a reduction in size of the apical radiolucent area (Figure 1F). Unfortunately, despite multiple attempts to locate the patient *via *cell phone and at her home address, it was not possible to obtain long term follow-ups in this case.

**Figure 2:**
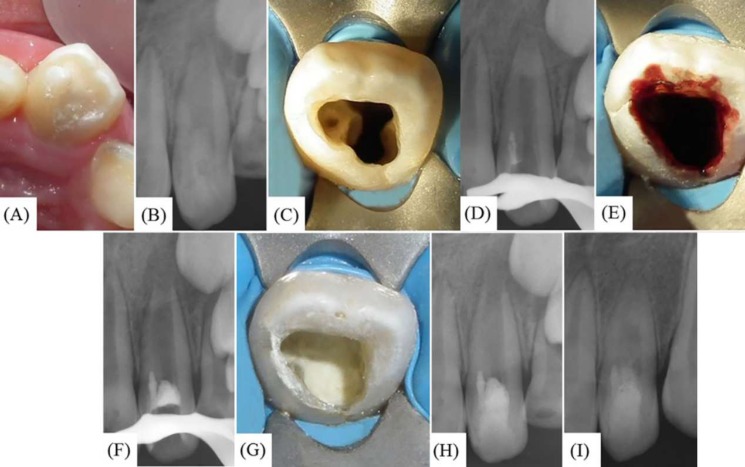
*A)* Maxillary left lateral incisor exhibits a cone-shaped morphology; *B)* Pre-operative radiograph showing Oehlers type II DI; *C) *Access cavity depicting two canal orifice entrances; *D)* Radiographic verification of mineral trioxide aggregate (MTA) placement in the invagination; *E)* Blood clot formation; *F)* Radiographic verification of MTA placement in the main canal; *G)* Clinical photograph of white MTA placement in the main canal; *H)* Immediate postoperative radiograph of revascularization technique; *I)* 32 month follow-up with radiographic evidence of notable increase in the thickening of the apical radicular walls, an incrementing in the root length, and resolution of the periapical radiolucency

***Case 2***: An 11-year-old male patient with no known medical history of disease and no known allergies was referred to the Department of Endodontics, University of Guadalajara, México, for endodontic treatment of a maxillary left lateral incisor. The patient complained of intense spontaneous pain for the previous five days and was able to indicate the source of his discomfort as tooth #22. Clinically, the crown of the maxillary left lateral incisor was cone-shaped without caries (Figure 2A). The tooth was extremely sensitive to percussion and palpation, with grade one mobility. The tooth responded negatively to pulp sensitivity testing with Endo-Ice™ (Hygenic Corp., Akron, OH, USA) and to the electric pulp tester. Periodontal probing depths were within normal limits. A periapical radiograph revealed a type II invagination extending from the crown to the middle root, with communication to the main canal and adjacent radiolucent area. Moreover, a wide open and divergent apical foramen with nearly completed root formation was detected (Figure 2B). No history of previous trauma was reported. An endodontic diagnosis of pulp necrosis with acute apical abscess was made. The following treatment options were discussed with patient’s parents: *a)* endodontic therapy followed by apical plug with mineral trioxide aggregate (MTA), *b)* revascularization, or *c)* extraction. Given the immature stage of root development of the affected tooth, the revascularization option was recommended. 

After obtaining written informed consent from the patient’s parents, local anesthesia was administered using 1.8 mL 2% lidocaine 1:100,000 epinephrine (Alphacaine 100; DFL, Rio de Janeiro, RJ, Brazil) and the tooth was isolated with a dental dam. A surgical operating microscope was utilized during the entire clinical procedure. Endodontic access was performed using a high speed diamond bur and two canal entrances were located (Figure 2C). One of the canal entrances corresponded to the main canal and the other to the invagination. The access was redefined with ultrasonic tips START-X #3 and #4 (Dentsply Maillefer, Ballaigues, Switzerland) and the working length was radiographically corroborated. The internal canal walls were minimally instrumented using hand K-files (Dentsply Maillefer, Ballaigues, Switzerland) and irrigated with 20 mL of 3.0% NaOCl, and then gently dried with paper points. After that, calcium hydroxide paste (a creamy paste made of equal proportions of calcium hydroxide powder and saline solution) was delivered into the two canals with a lentulo spiral instrument, and then the tooth was temporized with Cavit G (3M ESPE, St Paul, MN; USA) for 14 days.

At the following appointment, the patient was asymptomatic and local infiltration was administered. After dental dam placement and access into the canals, the intracanal dressing was removed with copious irrigation of 10 mL of 1% NaOCl and hand K-files of small sizes. Subsequently, a sterilized cotton pellet was placed at the entrance of the main canal; the invagination was filled with white MTA (ProRoot MTA, Dentsply Tulsa Dental, Tulsa, OK; USA) with the aid of a specialized metal carrier (MAP System, Universal Kit, Dentsply, Switzerland) and sequential compaction using Schilder pluggers (Dentsply, Maillefer, Switzerland). Proper compaction was radiographically verified (Figure 2D). The cotton pellet was then removed from the main canal entrance and the canal was dried with paper points. Bleeding was induced by over-instrumenting into the periapical tissues with a #25 pre-curved hand K-file (Figure 2E). Once the coagulum was formed at the level of the cemento-enamel junction, a 3 mm thick barrier of white MTA was placed on top of the coagulum (Figures 2F and 2G) followed by a wet cotton pellet on top of the MTA to allow for proper setting of the material. The tooth was temporized with glass ionomer cement. The patient was prescribed 250 mg of Paracetamol. One day after the wet cotton pellet was removed, the setting of the MTA was confirmed and the access opening was sealed with Filtek Supreme XT composite resin (3M ESPE, St Paul, MN). A final radiograph was performed for verification purposes (Figure 2H). At the 32 month follow-up, the patient continued to be asymptomatic. Upon clinical examination, tooth #22 was functional with normal periodontal condition, non-responsive to cold and slight change in coloration of the remaining dental structure. The radiographs showed evidence of complete resolution of the pre-existing apical radiolucency, apical closure, thickening of the root canal walls and increase in root length (Figure 2I).

## Discussion

The cases here presented illustrate the successful non-surgical management for type II DI with periapical lesions, one with a closed apex and one with an open apex. Conservative endodontic treatment should be considered as the first line of treatment rather than periapical surgery or other more invasive procedures, such as intentional replantation or extraction and implant placement. In cases with immature roots, the use of proper intracanal disinfection methods and revascularization protocols can be successful. 

Of the various types of DI reported in the literature, Kristoffersen *et al.* [13] stated that the Oehlers type II represents the most clinically challenging variation for the clinician, because the invagination usually obstructs the ideal access path to the canal system and thus complicates adequate chemo-mechanical instrumentation and three-dimensional filling. The complexity of the canal anatomy found in these cases, particularly between the main canal and the invagination, may prevent the direct contact of antibiotics or intracanal medications such as calcium hydroxide, with the canal.

Preventive restorations with resin composites or fissure sealants have been advocated for type II DI cases in which the pulp is still vital. According to Zhu *et al.* [1] this method should the first choice of treatment in order to prevent possible complications, such as pulp necrosis. However, Ridell *et al.* [14] reported a failure rate of 11% in the teeth that had undergone prophylactic DI treatment, and all failed cases were teeth with DI type II.

In cases with type II DI and pulpal necrosis, in which the invagination is close to the cemento-enamel junction, the main canal can be treated without removing the invagination, as demonstrated in case 1. Although no further weakening of the root occurred by preserving the invagination, it has been speculated that the untouched canal walls may harbor bacterial remnants or biofilms [15]. Therefore, in case 1 ultrasonic irrigation and a final rinse of 17% EDTA were utilized as an adjunct after the cleaning and shaping process. Studies have shown that such strategies remove significantly more smear layer or bacteria from the root canal [16]. In contrast, if the DI extends into the middle or apical third of the root, it could be carefully removed with the aid of the surgical operating microscope and ultrasonic techniques [12, 17], as was described in case 2.

Teeth with open apices and thin root canal walls complicate conventional endodontic treatment, because of an unfavorable crown/root ratio, thin dentinal walls that are susceptible to fractures, and lack of apical stop that would prevent the root filling material from invading periodontal tissues [18]. In such cases, apexification procedures are the method of choice [1]. However, the use of conventional apexification has several disadvantages, such as long treatment intervals, risk of re-infection of the root canal system between visits, possible negative effects of long-term calcium hydroxide treatment, which increases the fracture-resistance of roots and does not promote continued root development [19, 20]. Due these limitations, the revascularization technique has been considered an alternative of treatment for the management of DI immature teeth with necrotic pulps [21-24].

The revascularization procedure in case 2, was performed according to the American Association of Endodontist (AAE) [25] and European Endodontic Society (ESE) [26] guidelines, which are based in three important principles [27]: *a)* chemo-mechanical elimination of bacteria with minimal to no instrumentation from the canal system, followed by calcium hydroxide intra-canal dressing, *b)* evoking bleeding from periapical tissues, which acts as a scaffold for the ingrowth of new tissue, and *c)* prevention of reinfection by creating a bacteria-tight seal with a bioactive material. As a result, several radiographic outcomes could be detected, such as the resolution of apical lesions, increasing the thickness of the radicular walls, incrementing the root length and apical closure [28] (Figure 2I). All these are significant benefits in teeth at the early stage of root development, mainly if the lumen of the apex shows a larger diameter that the lumen of the root canal, as was seen in case 2. Regardless of the clinical protocol applied during the revascularization technique, evidence suggests that the healing is always the same. Human and animal studies have confirmed that the new hard tissue formed is not a regeneration of the pulp-dentin complex [29-32]. A mix bone and cement comprised the reparative tissue deposited in the pulpal space. 

## Conclusion

Early detection of teeth with DI type II and proper exploration of their internal anatomy are key factors for their successful management. As demonstrated in this report, conservative non-surgical endodontic treatment should be the first line of treatment for these cases. The use of revascularization protocols in teeth with pulp necrosis and early stage of root development could be a better alternative than traditional apexification techniques.
